# Prevalence and Risk Factors of Group B *Streptococcus* Colonization in Pregnant Women: A Pilot Study in Palestine

**DOI:** 10.1155/2021/8686550

**Published:** 2021-12-13

**Authors:** Mohammad Qadi, Adham AbuTaha, Ro'ya Al-Shehab, Salsabil Sulaiman, Abdallah Hamayel, Amjad Hussein, Shatha AbuTaha, Ayman Dawoud, Faizeh Hussein

**Affiliations:** ^1^Department of Biomedical Sciences, Faculty of Medicine and Health Sciences, An-Najah National University, Nablus, P. O. Box. 7, State of Palestine; ^2^An-Najah National University Hospital, Nablus, P. O. Box. 7, State of Palestine; ^3^Department of Medicine, Faculty of Medicine and Health Sciences, An-Najah National University, Nablus, P. O. Box. 7, State of Palestine

## Abstract

**Background:**

Maternal *Streptococcus agalactiae* (Group B *Streptococcus* (GBS)) colonization is an important cause of complications in mothers and neonates during gestation and after delivery. The data regarding GBS colonization among pregnant women in Palestine is scarce. The aim of this study is to determine the prevalence of GBS colonization, its associated risk factors, and the antibiotic sensitivity patterns in Nablus, West Bank, Palestine.

**Methods:**

A cross-sectional, single center study conducted at Rafidia Governmental Hospital in Nablus, West Bank, Palestine. Samples were collected between November 2019 and January 2020. Vaginal swabs from 200 pregnant women (≥35 weeks of gestation) attending the labor and delivery department were plated directly on CHROMagar^TM^ StrepB (CHROM agar, France) and placed in an incubator at 35–37°C. After 24 and 48 hours, the plates were checked for growth and classified into three categories: growth of GBS with mauve colonies on chromogenic media, no growth, or other growth. The identification of the mauve colonies was confirmed by the CAMP test. Identified GBS isolates were tested for susceptibility to vancomycin, ampicillin, clindamycin, cefotaxime, erythromycin, and levofloxacin using the disc diffusion method. Clinical and demographic information were collected using a questionnaire.

**Result:**

The overall prevalence of GBS colonization was 12%. The median age of the study population was 27 years. GBS colonization was significantly associated with age (*p*=0.013), history of previous preterm delivery (*p*=0.013), and parity (*p*=0.015). No association was noted with smoking, previous abortion, previous history of fetal demise, vaginitis, or urinary tract infection. Resistance to ampicillin, vancomycin, cefotaxime, erythromycin, clindamycin, and levofloxacin was found to be 91.7%, 54.2%, 45.8%, 29.2%, 25%, and 8.3%, respectively.

**Conclusion:**

The prevalence of vaginal GBS in this study was 12% from Nablus, West Bank. Further research is needed to determine the GBS serotypes common in West Bank and the burden they cause on the health system. Moreover, this study also highlights the need to establish a screening program suited to a developing country with low control on the antibiotic's prescription protocols.

## 1. Introduction

*Streptococcus agalactiae*, also known as Group B *Streptococcus* (GBS), is *β*-hemolytic Gram-positive cocci. It can be a constituent of the human normal flora [1], found asymptomatically in the maternal genital and gastrointestinal tracts. It is estimated that 18% of women worldwide are colonized with GBS [2].

GBS colonization in pregnant women has a significant clinical burden, and it has been identified as the primary risk factor for the development of early-onset GBS disease [3]. GBS can cause ascending infections which may lead to serious consequences for the mother and the baby during gestation and after delivery, including maternal sepsis, stillbirth, and neonatal death [4–6]. Around 4 out of every 10,000 pregnant women have GBS sepsis during their pregnancy [6], and a study in Ireland showed that GBS accounted for 25% of clinically significant bacteremia in hospitalized pregnant women [7]. Moreover, it has been reported that 1% of all stillbirths in developed countries and 4% of stillbirths in Africa are associated with GBS [5], and the odds of having a stillbirth in Eastern Ethiopia are 8.93 times higher among GBS-colonized pregnant women [8]. Worldwide, the case fatality rate for infants with GBS is estimated to be around 8.4% [4].

GBS is the most common cause of early-onset neonatal sepsis and meningitis [9, 10], and is also a major cause of preterm labor, premature rupture of membranes, chorioamnionitis, and bacteremia [10]. GBS colonization is also a known risk factor for the development of late-onset GBS infections, which commonly manifest as bacteremia without an apparent focus of infection [11]. These late-onset infections may also cause meningitis and may rarely lead to focal infections affecting bones, joints, soft tissue, or the urinary tract.

There are multiple risk factors associated with GBS colonization, including older maternal age, lower gestational age, length of premature rupture of membranes, midwife delivery, and experiencing meconium-stained amniotic fluid [12, 13].

The Centers for Disease Control and Prevention (CDC) recommends that all pregnant women between the 35^th^ and 37^th^ gestational weeks should be screened for vaginal-rectal GBS colonization and that those with positive swab cultures should receive intrapartum antibiotic prophylaxis (IAP). In addition, pregnant women with a previous infant with GBS disease, GBS bacteriuria in the current pregnancy, as well as those with unknown colonization status and intrapartum risk factors such as maternal fever or prolonged rupture of membranes are provided with IAP during labor [14]. In the absence of IAP, it is estimated that about 50% of newborn infants born to women colonized with GBS become colonized with GBS themselves, and of those, 1–2% will develop early-onset GBS disease [15].

The gold standard method for diagnosis of GBS colonization comprises culture of combined vaginal and rectal samples in a selective enrichment medium. There are two types of selective media for GBS, the Granada (anaerobic) medium and the ChromID^TM^ Strepto B medium (aerobic) (CHROM-B). Recent developments of real-time polymerase chain reaction (PCR) and fluorescence labelling technologies have provided new methods for bacterial identification [16]. GBS-specific PCR assays are highly sensitive and specific, but they are expensive [17].

In Palestine, there is no screening program for GBS colonization among pregnant women. To the best of our knowledge, there are no previous studies about Palestinian GBS colonization, except for a study in the Gaza Strip [18]. The objective of this study was to estimate the prevalence of GBS colonization in the city of Nablus, the associated risk factors for colonization, and the antibiotic susceptibility of the GBS isolates.

## 2. Materials and Methods

### 2.1. Study Design

A cross-sectional study was conducted over a period of 3 months.

### 2.2. Study Setting and Population

Parturient women attending Rafidia Governmental Hospital for vaginal delivery in the period extending from November 2019 to January 2020 were invited to participate in the study. Rafidia Hospital is the only major obstetric governmental hospital in Nablus, Palestine, with about 450–550 pregnant women admitted for delivery each month. Pregnant women on any antibiotic treatment in the last two weeks prior to data collection and those less than 35 weeks of gestation were excluded.

### 2.3. Questionnaire

Ladies who agreed to participate were interviewed face-to-face with a structured questionnaire which was prepared in the local language (Arabic). The questionnaire included gestational and demographic variables like maternal age, employment status, smoking, family income, place of residence, parity, and other pregnancy-related characteristics like previous miscarriages, preterm labor, vaginal discharge, vaginal pruritus, vaginal burning, vulvitis, and vaginal candidiasis.

### 2.4. Sample Collection

Each swab was brushed against the posterior vaginal wall with the patient in the lithotomy position without the use of a speculum.

### 2.5. Isolation of GBS

The swabs were plated directly on CHROMagar^TM^ StrepB (CHROMagar, France) and placed in an incubator at 35–37°C. After 24 and 48 hours, the plates were checked for growth and classified into three categories: growth with mauve colonies on chromogenic media, no growth, or other growth. GBS identification was confirmed by performing a CAMP test on all growth with mauve colonies. A single straight-line streak of beta-hemolysin producing *Staphylococcus aureus* was made on a blood agar plate. Subsequently, a perpendicular streak of the beta-hemolytic *Streptococcus* was added to be identified. Then, the plates were incubated at 35°C in ambient air for 18–24 hours. Plates were observed for areas of increased hemolysis at the intersection of both streaks. The beta-hemolysin secreted by *Staphylococcus* and the CAMP factor secreted by group B *Streptococcus* resulted in an increased area of hemolysis.

### 2.6. Antibiotic Susceptibility

All confirmed GBS isolates were subcultured onto blood agar plates for 24 hours at 37°C, and tested for susceptibility to vancomycin, ampicillin, clindamycin, cefotaxime, erythromycin, and levofloxacin using BD BBL™ Sensi-Disc™ dispensers according to the CLSI Performance Standards for Antimicrobial Susceptibility Testing [19]. All recommended antibiotics from group A and group B were tested, in addition to one antibiotic from group C. Ampicillin was used as an alternative to penicillin in susceptibility testing, in compliance with the aforementioned guidelines.

### 2.7. Statistical Analysis

Results were analyzed using the IBM Statistical Package for Social Sciences program (SPSS) version 21. The relationships between categorical variables were analyzed using the Chi-square test for small cell sizes (*n* < 5). A *p* value of less than 0.05 was considered significant. For continuous variables, data were expressed as median ± SD. Categorical variables were expressed as frequencies and percentages.

### 2.8. Ethical Considerations

Approval was obtained from the Institutional Review Board (IRB) of An-Najah National University and from the Ministry of Health. A written informed consent was obtained from all participants.

## 3. Results

### 3.1. Patient Characteristics

200 women who presented with labor between November 2019 and January 2020 to Rafidia Governmental Hospital participated in the study; their demographic and clinical characteristics are displayed in Table 1. The median age of participants was 27 years, 61.5% were city residents, and about half (58%) of them had an income between 3500 and 5000 shekels. Two-thirds (76%) of the women had been pregnant before, and a few (8.5%) reported cigarette smoking.

### 3.2. GBS Colonization

The overall prevalence of GBS colonization as determined by chromogenic culture and confirmed by the CAMP test was 12%. Most (83.3%) of the colonized women were between 25 and 33 years old, while 4.2% were younger than 25 years and 12.5% were older than 33 years. The frequency of GBS colonization among different age groups was statistically significant (*p* < 0.05). The age groups of GBS-colonized and noncolonized women are shown in Table 2.

### 3.3. Risk Factors

Different factors associated with GBS colonization are outlined in Table 3. There was no significant difference between GBS-colonized women and noncolonized women in terms of symptoms of vaginal infections (*p* > 0.05) nor in symptoms of urinary tract infections (*p* > 0.05). In addition, there was no significant difference in smoking status between the two groups (*p* > 0.05).

This study's data showed a significant difference regarding previous preterm delivery (*p*=0.013) and previous pregnancy (*p*=0.015). However, no statistically significant difference was observed with a history of previous abortion in the first or second trimester (29.9% and 0.0%, respectively) or with fetal demise (4.2%) (*p* > 0.05).

### 3.4. Antimicrobial Susceptibilities of GBS

Of the 24 examined GBS isolates, 16 (66.7%) were susceptible to levofloxacin, 14 isolates (58.3%) to clindamycin, 13 (54.2%) to cefotaxime, 11 (45.8%) to each vancomycin and erythromycin, and 2 (8.3%) to ampicillin, as shown in Table 4.

## 4. Discussion

### 4.1. Prevalence of GBS among Pregnant Women

GBS colonization in pregnant women carries a significant clinical burden, as it is associated with maternal and fetal morbidity and mortality. One meta-analysis showed that overall, 18% of women worldwide are colonized with GBS, with regional variation in prevalence (11%–35%) [20]. In this study, the prevalence of GBS colonization was 12%, similar to another study conducted in southern Israel which showed a prevalence of 12.3% [21], while other studies conducted in Gaza and Nazareth showed a higher prevalence of GBS colonization (21% and 31%, respectively) [18, 22]. Similarly, surrounding countries had a higher prevalence of colonization; 27.4%, 19.5%, and 18.4% in Egypt [23], Jordan [24], and Lebanon [25], respectively. The differences in the colonization rate could be attributed in part to specimen collection methods and lab-related causes.  Table 5 summarizes the methods used in the studies describing the GBS colonization rate in the vicinities of the West Bank.

The prevalence rates of GBS colonization vary in different geographical regions across the globe. This study showed similar results to those of other studies in Saudi Arabia [26], Tunisia [27], and China [28]. However, studies from Kuwait [29], Morocco [30], Iran [31], Turkey [32], and Ethiopia [33] reported higher prevalence rates than those found in this study. Meanwhile, on the other hand, lower prevalence rates were reported in India [34] and South Korea [35].

GBS colonization rates are affected by a number of patient and environmental characteristics, including race, origin, age, parity, socioeconomic status, diet, climate, and maternal hygiene. Colonization rates are also affected by the medium used and the number and type of sites that have been cultured [36, 37]. Using vaginal swabs only as a collection method has been shown to lower the prevalence of GBS colonization for the same population. This was exemplified in a study in Ethiopia, in which the overall carriage rate was 19% while the vaginal carriage rate was 10.4% [33]. In addition, in a study in Iran, the frequency of GBS-positive culture results from rectal samples was higher than that using vaginal samples only [31]. After initial detection of GBS colonies, the confirmation method also seems to play a role in the reported GBS colonization rate. That is, the more specific the confirmation method, the lower the reported GBS colonization rate, and the more sensitive the confirmation method, the higher the reported GBS colonization rate. For instance, a study in Iran mentioned that the use of PCR confirmed higher positive results for GBS than culture confirmation techniques due to the high sensitivity of PCR [31].

Although culturing on CHROMagar followed by CAMP test confirmation gave the comparable prevalence of 12%, the PCR technique may fine the prevalence as it is a more sensitive technique. In addition, one source of the samples from vaginal swamps may underestimate the prevalence that may be modified with the simultaneous vaginal and rectal samples; therefore, the prevalence may be greater than that obtained from vaginal samples only.

### 4.2. Risk Factors

In this study, women with a history of preterm delivery had a significantly higher frequency of GBS colonization. Similar findings have been reported in studies conducted among Danish women [38] and Canadian women [39]. This is presumably because women colonized with GBS at delivery are more likely to have a preterm delivery than women who were not colonized [38]. In contrast, studies among Australian women showed no association between preterm delivery and GBS colonization [40].

With the exception of age and parity, no statistically significant association was observed between GBS colonization and any sociodemographic characteristics in our study. This may be in part due to the small sample size and the large number of variables included as sociodemographic characteristics. These results are similar to a study conducted in Brazil, which concluded that GBS colonization does not appear to be directly related to socioeconomic factors [41], and a study conducted in Iran [42], where there were no significant differences in colonization rates based on age or parity. In contrast, increasing age or parity in some studies in Tanzania has been shown to be associated with increased GBS colonization [43].

Contrary to our results, an Australian study [44] demonstrated an association between GBS colonization and spontaneous abortions. Meanwhile, our findings confirm those of another study in Tanzania [43], in which there was no association found between GBS colonization rate and spontaneous abortions.

While our study found no association between urinary tract infections during pregnancy and GBS colonization, a study from the Democratic Republic of the Congo found that they were associated with a high odds ratio for vaginal colonization [45], similar to results found in Korea [46].

We also reported no association between GBS colonization and vaginitis, in contrast to a Tunisian study [47]. Moreover, no association between colonization with GBS and smoking was found in this study, while studies in Iran [48] and the USA [49] demonstrated an association. Numerous factors play a role in these differences of reporting associations in each study, and we suspect that a small sample size, among other variables, contributes to this difference.

### 4.3. Antibiotic Sensitivity

Antibiotic resistance is a growing problem worldwide, and GBS is no exception. In the present study, the rate of resistance to ampicillin was very high (91.7%), while mildly reduced susceptibility to ampicillin was found in a study in Ethiopia [50], and no resistance to ampicillin was found in studies conducted in Saudi Arabia [26] and Cameroon [36].

High rates of vancomycin resistance have been detected in our study (54.2%). In contrast, 100% susceptibility was found in other studies conducted in the USA [51] and in Brazil [52].

The high resistance of GBS isolates in our study to ampicillin and vancomycin is possibly because of the widespread empiric use of antibiotics for the treatment of different infectious diseases, and the availability of these drugs nonrestrictively and inexpensively in different areas enables self-prescription.

In contrast to our study, where 45.8% of GBS isolates were resistant to cefotaxime, 100% susceptibility was detected in studies conducted in the USA [53] and in Brazil [54]. Moreover, resistance in our study to erythromycin and clindamycin was 29.2% and 25%, respectively, which is significantly higher than recent studies conducted in Kuwait [55] and in Tanzania [43]. As for levofloxacin, it had the highest susceptibility rate in this study, closer to the results documented in a study in Argentina [56].

## 5. Conclusion

This research was the first to be conducted in the West Bank regarding GBS colonization in pregnant women. The prevalence of GBS in this study was 12%, and further research is needed to determine the common GBS serotypes in the West Bank and the burden they may cause on the health system. This study also highlights the need to establish a screening program suited to a developing country, with consideration given to the growing problem of antibiotic resistance.

### 5.1. Limitations

- Most participants refused rectal swabs, so only vaginal swabs were obtained
- As a cross-sectional study, the causal relationship cannot be assessed
- The study did not assess the serotypes of GBS in colonized women

## Figures and Tables

**Table 1:** Sociodemographic characteristics of participants.

Characteristics	*N* (%)
Age (years)	27^a^ (8)^b^
16–24 years	52 (26%)
25–33 years	125 (62.5%)
34 years and older	23 (11.5%)

Income (Israeli shekels)	
** **<2500	4 (2%)
** **2500–3500	72 (36%)
** **3500–5000	116 (58%)
** **>5000	8 (4%)

Residence	
** **City	123 (61.5%)
** **Village	77 (38.5%)

Prior history of pregnancy	
** **Yes	152 (76%)
** **No	48 (24%)

Smoker	
** **Yes	17 (8.5%)
** **No	183 (91.5%)

^a^Median; ^b^standard deviation.

**Table 2:** Age groups of GBS-colonized and noncolonized women.

	GBS-colonized women	Noncolonized women	*p* value
Age (years)	28^a^ (7)^b^	27^a^ (8)^b^	0.031
16–24 years	1 (4.2%)	51 (29%)	0.009
25–33 years	20 (83.3%)	105 (59.7%)	0.025
34 years and older	3 (12.5%)	20 (11.4%)	0.87

^a^Median; ^b^standard deviation.

**Table 3:** The medical history of both GBS-colonized and noncolonized pregnant women.

	GBS-colonized women (*N*) (%)	Noncolonized women (*N*) (%)	*p* value
Smoker	3 (12.5%)	14 (8%)	0.436
Previous pregnancy	23 (95.8%)	129 (73.3%)	0.015
One pregnancy	7 (29.16%)	26 (14.8%)	0.026
2–3 pregnancies	7 (29.16%)	53 (30.1%)	0.981
≥4 pregnancies	9 (37.5%)	50 (28.4%)	0.744
Previous preterm^a^	5 (20.8%)	11 (6.3%)	0.013
Number of abortions			
** **0	16 (66.7%)	144 (81.8%)	0.082
** **1	7 (29.2%)	29 (16.5%)	0.129
** **≥2	1 (4.2%)	3 (1.7%)	0.403
Time of abortion			
** **First trimester^b^	7 (29.2%)	35 (19.9%)	0.295
** **Second trimester^c^	0 (0.0%)	8 (4.5%)	0.599
** **Fetal demise^d^	1 (4.2%)	1 (0.6%)	0.226
UTI symptoms^e^	8 (33.3%)	44 (25%)	0.383
Vaginal infection symptoms ^f^	7 (29.2%)	40 (22.7%)	0.485

UTI: urinary tract infection, abortion: fetal death before 24 weeks of gestation, ^a^delivery before 37 weeks of gestation, ^b^the first 12 weeks of gestation, ^c^from 13 weeks to 28 weeks of gestation, ^d^death of a fetus after 24 gestational weeks, ^e^urinary frequency, urgency, dysuria, ^f^increased or foul-smelling vaginal discharge, burning, or itching.

**Table 4:** Antibiotic susceptibilities of 24 GBS isolates from colonized pregnant women.

Antibiotic	Sensitive, *N* (%)	Intermediate, *N*(%)	Resistant, *N* (%)
Erythromycin	11(45.8%)	6 (25%)	7 (29.2%)
Ampicillin	2 (8.3%)	—	22 (91.7%)
Vancomycin	11(45.8%)	—	13 (54.2%)
Levofloxacin	16 (66.7%)	6 (25%)	2 (8.3%)
Cefotaxime	13 (54.2%)	—	11 (45.8%)
Clindamycin	14 (58.3%)	4 (16.7%)	6 (25%)

**Table 5:** Studies describing the GBS colonization rate in the vicinities of the West Bank.

First author	Year	Location	Sample population	Sample size	Collection method	Confirmation method	Colonization rate (%)
Qadi (this study)	2020	Nablus, West Bank, Palestine	Pregnant women (>35 weeks' gestation)	200	Vaginal	CAMP test	12
Marchaim et al. [21]	2003	Southern Israel	Pregnant women (>35 weeks' gestation)	681	Vaginal and rectal	CAMP test and agglutination test	12.3
Nabil et al. [18]	2017	Gaza, Palestine	Pregnant women (>35 weeks' gestation)	200	Vaginal and rectal	PCR test	21
Clouse et al. [24]	2019	Jordan	Pregnant women (>35 weeks' gestation)	200	Vaginal and rectal	Latex agglutination	19.5
Hakim et al. [22]	2018	Nazareth Arab-Israeli	Pregnant women (>35 weeks' gestation)	188	Vaginal and rectal	(AmpliVue® GBS assay), atoB gene	31
Shabayek et al. [23]	2013	Egypt	Pregnant and nonpregnant women	364	Vaginal	PCR	27.4
Ghaddar et al. [25]	2014	Lebanon	Pregnant women (>35 weeks' gestation)	168	Vaginal	Latex agglutination	18.4

## Data Availability

The data used to support the findings of this study are included within the article.
